# A framework to assess the added value of subgroup analyses when the overall treatment effect (TE) was significant

**DOI:** 10.1186/1745-6215-14-S1-P32

**Published:** 2013-11-29

**Authors:** Hong Sun, Werner Vach

**Affiliations:** 1 Institute of Medical Biometry and Medical Informatics, Freiburg, Germany

## 

Although statisticians have already awared and discussed the problem, it is still not clear whether we should perform and how to perform such subgroup analyses when the overall TE is significant in both single clinical trials and systematic reviews. So a framework is needed to assess and compute the long term effect of different strategies to perform subgroup analysis.

We propose two performance measures to evaluate the average post-study TE for patients in all studies (E) and fraction of patients with a negative TE in the positive studies (P). Nine existing decision rules are applied to different assumptions of subgroup specific and individual TE. Optimistic, moderate and pessimistic scenarios are assumed for true TE.

A hierarchical structured simulation study is set up with the fixed power of 90% and an assumed constant TE for each study in particular case of binary outcomes with TE presented as risk difference, a constant sample size 280 is required. Patients were allocated in 1:1 ratio to two hypothetical treatment subgroups. 10000 simulation runs applied to each scenario.

We demonstrate that there are decision rules for subgroup analysis which decrease P and increase E simultaneously comparing to the situation of no subgroup analysis. These rules are much more liberal than the usual significance testing, since there is a high risk to decrease E using the latter.

